# Optimal parameters for determining the LH surge in natural cycle frozen-thawed embryo transfers

**DOI:** 10.1186/s13048-017-0367-7

**Published:** 2017-10-16

**Authors:** Mohamad Irani, Alex Robles, Vinay Gunnala, David Reichman, Zev Rosenwaks

**Affiliations:** 1 The Ronald O. Perelman and Claudia Cohen Center for Reproductive Medicine, Weill Cornell Medicine, 1305 York Avenue, New York, NY 10021 USA; 2 Department of Obstetrics and Gynecology, Weill Cornell Medicine, New York, NY 10021 USA

**Keywords:** LH surge, Natural cycle frozen-thawed embryo transfer, Luteinizing hormone, Estradiol, Timing of embryo transfer

## Abstract

**Background:**

There is no consensus on the exact parameters that define the LH surge for natural cycle frozen-thawed embryo transfers (NC-FET). Accurately determining the LH surge would affect the timing, and subsequently the success rates, of embryo transfer. Therefore, the aim of this study was to delineate the optimal levels and relationship for luteinizing hormone (LH) and estradiol in an effort to optimally identify the LH surge in NC-FET.

**Methods:**

It is a retrospective study that was performed in an academic medical center. Patients who underwent blastocyst NC-FET who either had preimplantation genetic screening (PGS) or were <35 years old but did not undergo PGS (non-PGS) were included in separate analyses. They were divided into two groups: Group A included patients whose LH surge was defined as the first attainment of LH ≥ 17 IU/L during the follicular phase with a ≥30% drop in estradiol levels the following day; group B encompassed patients whose LH level continued to rise and the surge was defined as the highest serum LH level occurring a day after LH ≥ 17 IU/L despite a ≥ 30% drop in estradiol levels. The main outcomes measures were implantation and live birth rates.

**Results:**

Four hundred-seven non-PGS and 284 PGS NC-FET were included. Among non-PGS cycles, group A was associated with significantly higher implantation rates (48.7% vs. 38.1%) and live birth rates (52.9% vs. 40.1%) compared to group B. In contrast, group A and B had comparable live birth rates among PGS cycles.

**Conclusions:**

Among non-PGS cycles, measuring LH and estradiol levels the day after an LH ≥ 17 IU/L and defining the surge as the first day of LH ≥ 17 IU/L in the context of a ≥ 30% drop in estradiol the following day was associated with better NC-FET outcomes than defining the surge as the day representing the highest serum LH level despite a ≥30% drop in estradiol levels.

## Background

The trend towards transferring fewer embryos during fresh IVF cycles has prompted a substantial increase in the number of frozen-thawed embryo transfer cycles (FET) [1]. Additionally, FET after cryopreservation of all embryos has been widely used either to perform preimplantation genetic screening (PGS) or to minimize the risk of developing ovarian hyperstimulation syndrome (OHSS) [2]. FET can be accomplished with various endometrial preparation protocols including programmed cycles involving the use of exogenous estrogen and progesterone, or using the patient’s natural cycle and associated endogenous hormones [3]. NC-FET is considered by many clinicians the preferred method for women with regular ovulatory menstrual cycles because it does not require long-term exogenous hormonal replacement [4].

Blastocyst grading, ploidy status, endometrial thickness, and transfer technique affect FET outcomes [5–8]. In addition to these important factors, transfer of the thawed embryo(s) should be conducted at the time of optimal endometrial receptivity, which is determined by the time of luteinizing hormone (LH) surge during NC-FET. Currently, there is no consensus on the exact parameters that define the LH surge for NC-FET. If the LH surge is called too early, there is a risk of embryo-endometrial dysynchrony on transfer day. Conversely, defining the surge too late risks placing an embryo in an endometrium that has already advanced beyond the implantation window. With this in mind, we set out to analyze our historical data to examine the heterogeneity of defining the LH surge, and to determine whether we could identify optimal parameters to guide decisions regarding transfer timing.

## Methods

### Cycle inclusion criteria

The institutional review board at Weill Cornell Medical College approved this study. All NC-FET performed at our center from January 2010 through December 2015 were reviewed for potential inclusion. Patients who underwent blastocyst NC-FET who either had PGS or were younger than 35 years old but did not undergo PGS (non-PGS) were included in separate analyses. The exclusion criteria were recipients of donated oocytes and women who had a history of ashermans syndrome.

### Clinical protocols

Controlled ovarian hyperstimulation, trigger injection (hCG, GnRH agonist, or both), oocyte retrieval, embryo culture, cryopreservation, and embryo transfer were conducted according to our standard protocols [9]. Patients were stimulated with gonadotropins (Menopur [Ferring]; Gonal-F [EMD-Serono]; and/or Follistim [Merk]) followed by pituitary suppression with GnRH-antagonist (Cetrotide 0.25 mg [EMD-Serono] or Ganirelix Acetate 0.25 mg [Organon]). Alternatively, patients used GnRH-agonists (Lupron [Abbott Pharmaceuticals]) for pituitary suppression followed by stimulation with gonadotropins. Some women received pretreatment with E2 patches (Climara 0.1 mg, Bayer HealthCare) or birth control pills (Ortho-Novum, Janssen Pharmaceuticals) before initiating gonadotropins therapy.

Follicular maturation was triggered with human chorionic gonadotropin (Pregnyl [Schering-Plough]; Novarel [Ferring Phamaceuticals]; or Profasi [EMD-Serono]) when ≥ two follicles attained ≥17 mm. Women undergoing GnRH-antagonist cycles and deemed to be at high risk of developing OHSS received either 4 mg of Leuprolide Acetate or a combination of 4 mg of Leuprolide Acetate with 1500 IU of hCG to trigger follicular maturation. Oocyte retrieval was performed under conscious sedation 35–37 h following trigger. Fertilization was achieved via conventional IVF or intracytoplasmic sperm injection based on semen parameters and history of prior in vitro fertilization outcomes. Fresh embryo transfers were carried out either on day 3 or day 5 according to embryo quality, number, and clinical indication. Remaining viable embryos were examined for suitability for cryopreservation on day 5 and 6.

**Fig. 1:**
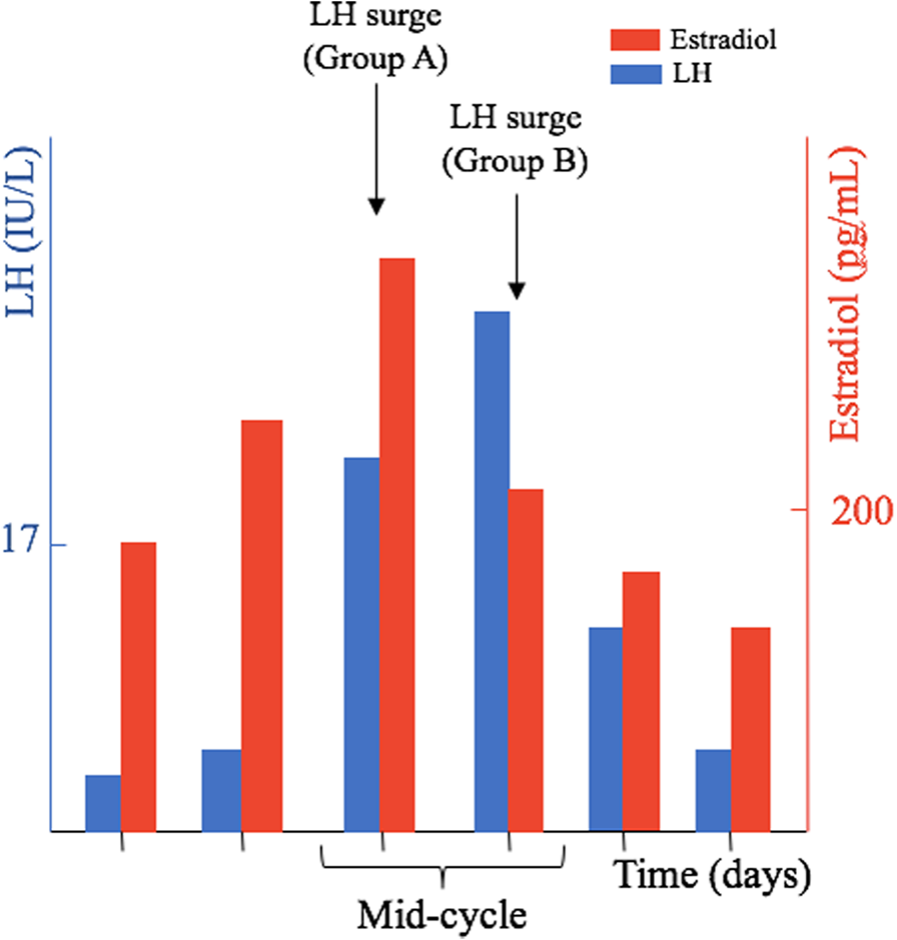
LH surge definition in both groups: Group A included patients whose LH surge was defined as the first attainment of LH ≥ 17 IU/L during the follicular phase with a ≥30% drop in estradiol levels the following day; group B encompassed patients whose LH level continued to rise and the surge was defined as the highest serum LH level occurring a day after LH ≥ 17 IU/L despite a ≥ 30% drop in estradiol levels. LH: Luteinizing hormone

Frozen-thawed embryo transfer was performed through a NC or programmed cycle. Only women undergoing NC-FET were included in this study. Patients in the non-PGS group underwent a prior fresh embryo cycle. During NC-FET, transvaginal ultrasonography was performed to assess follicle development and endometrial thickness/pattern beginning from cycle day 8–10 depending on the length of the patient’s menstrual cycle. Ultrasounds were repeated until a trilaminar lining >7 mm was attained. Starting 3–4 days prior to the suspected ovulation date, serum estradiol and LH levels were measured every 24 h until detecting the LH surge. The Siemens Immulite LH assay was used to measure serum LH levels. Practice heterogeneity due to physician preference in determining the LH surge occurred during the study period. Patients were divided into two groups depending on how the LH surge was defined: A) The first attainment of LH levels of ≥17 IU/L during the follicular phase with a drop in estradiol levels the following day; or B) rising LH level where the surge was defined as the highest LH level which occurred a day after LH of ≥17 IU/L despite a drop in estradiol levels (Fig. 1). These two groups were compared at three different levels of estradiol drop: ≥ 10%, ≥ 20%, and ≥30%. Blastocyst transfer was conducted 5 days following the determined LH surge (considering the LH surge as day 0 and transfer day as day 5) using Wallace catheters (Smiths Medical Inc., Norwell, MA).

### Laboratory procedures and blastocyst grading

All embryos were cultured individually in microwells of an EmbryoScope (Vitrolife, Sweden) with an integrated time-lapse system. Blastocysts were graded according to the three following morphological parameters: Inner cell mass (ICM), trophectoderm, and degree of expansion with hatching stage [5]. ICM morphology was graded into three categories: A- tightly packed cells; B- loosely packed cells; C- cells are not identifiable. Trophectoderm morphology was divided into three groups: A- many cells forming cohesive epithelial layer; B- cells of uneven size; C- few large cells squeezed to the side. The degree of expansion and hatching stage were the following: 1- The blastocoel is constituting <50% of the embryo; 2- the blastocoel is constituting >50% of the embryo; 3- the blastocoel is filling almost the whole blastocyst; 4- a very thin zona pellucida surrounding an expanded blastocyst; 5- the blastocyst is hatching; 6- the blasctocyst has completely hatched out of the zona pellucida. In order to adjust for blastocyst grading, which is a confounding variable, blastocysts were divided into four groups according to their morphological grading before cryopreservation: excellent (≥ 3AA), good (3-6AB, 3-6BA, 1-2AA), average (3-6BB, 3-6 AC, 3-6CA, 1-2AB, 1-2BA), and poor (1-6 BC, 1-6CB, 1-6CC, 1-2BB). Vitrification was performed on day 5 or day 6 based on the development of each embryo.

### Variables assessed

The primary outcome of this study was live birth rate (LBR) [10]. Secondary outcomes included spontaneous abortion and implantation rates. LBR was defined as the proportion of transfers resulting in live birth. Implantation rate (IR) was defined as the proportion of transferred embryos resulting in clinical pregnancy (intrauterine gestation identified by a transvaginal ultrasound). Spontaneous abortion rate was defined as the proportion of clinical pregnancies resulting in first trimester pregnancy loss. The following demographic characteristics were extracted: age, parity, body mass index (BMI), blastocyst grading, number of transferred embryos, and peak endometrial thickness.

### Statistics

All data analyses were conducted with STATA statistical software version 14 (StataCorp LP). Continuous variables were expressed as median [interquartile range]. They were compared by Mann-Whitney *U* test. Categorical variables were compared by Chi-square (*χ* ^2^) and Fisher’s exact tests. Odds ratios (OR) with 95% confidence intervals (CI) were calculated and adjusted for patient age, BMI, blastocyst grading, number of transferred embryos, and peak endometrial thickness using multivariate logistic regression. A value of *p* < 0.05 was considered statistically significant.

## Results

### NC-FET of women younger than 35 years old who did not undergo PGS

A total of 407 non-PGS NC-FET for women younger than 35 years old (*n* = 365 women) were included. Group A and B had comparable implantation (*p* = 0.6) and live birth rates (*p* = 0.5) when defining estradiol drop as ≥20%. Similarly, there was no difference between the two groups when using 10% as the threshold to define estradiol drop.

When considering estradiol drop as a drop of ≥30% in estradiol level, there were significant differences in NC-FET outcomes between group A (*n* = 270) and group B (*n* = 137). Demographic characteristics of all patients are summarized in Table 1. There were no significant differences in patient age, number of transferred embryos, blastocyst grading, infertility diagnosis, number of blastocysts cryopreserved, and peak endometrial thickness between group A and group B. The median BMI was higher in group A (23 [20.9–25.4] Kg/m^2^) compared to group B (21.6 [20.4–23.9] Kg/m^2^).

**Table 1:** Demographic characteristics of patients younger than 35 years old who did not undergo preimplantation genetic screening

Characteristic	Group A (*n* = 270)	Group B (*n* = 137)	*P* value
Age (years)	32 [30–33]	32 [31–33]	0.08
Parity	1 [0–1]	0 [0–1]	<0.001
BMI (kg/m^2^)	23 [20.9–25.4]	21.6 [20.4–23.9]	0.01
< 18.5	4.8%	8.0%	0.26
18.5–29.9	85.5%	83.3%	0.56
≥ 30	9.6%	8.7%	0.85
Peak endometrial thickness (mm)	8.7 [7.8–9.9]	8.6 [7.8–9.7]	0.79
Number of transferred embryos	1 (1–2]	1 [1–2]	0.49
Progesterone level on the day of the defined LH surge	0.8 [0.7–1.0]	1.0 [0.8–1.2]	<0.001
Blastocyst grading			
Excellent	0.4%	0.7%	1.0
Good	6.9%	8.6%	0.54
Average	79.5%	74.8%	0.35
Poor	13.1%	15.7%	0.52
Infertility diagnosis			
Male factor	40.3%	43.7	0.52
Ovulatory dysfunction	4.8%	3.6%	0.79
Diminished ovarian reserve	5.9%	6.5%	0.82
Tubal	11.1%	8.0%	0.38
Endometriosis	4.4%	5.1%	0.80
Uterine	1.1%	2.1%	0.40
Idiopathic	14.1%	14.5%	0.26
Other	18.1%	16.0%	0.48
Number of blastocysts cryopreserved	3 [0–5]	3 [2–5]	0.83
% IVF-ICSI	77.4%	76.6%	0.90

BMI: Body mass index. IVF-ICSI: in vitro fertilization with intracytoplasmic sperm injection. Values were expressed as median [interquartile range]

The number of transferred embryos and peak endometrial thickness did not affect the likelihood of LBR. Non-obese patients (BMI < 30 kg/m^2^) had a significantly higher LBR than obese patients (BMI ≥ 30 kg/m^2^) (*p* < 0.001). The odds ratio remained significant after adjusting for blastocyst grading and number of transferred embryos (adjusted odds ratio (aOR) = 6.2; 95% CI = 2.5–15.7). They also had a significantly higher implantation rate compared to obese patients (aOR = 2.8; 95% CI = 1.5–5.3). Obese patients had a significantly higher spontaneous abortion rate compared to non-obese patients (50% vs. 10.9%; *p* = 0.001; OR = 8.2; 95% CI = 2.4–28.0). The adjusted OR for spontaneous abortion was 8.4 (95% CI = 2.4–29.5) in the obese group compared to the non-obese group. After excluding obese patients, group A conveyed higher LBR compared to group B (56.1% vs. 44%; *p* = 0.02; OR = 1.7; 95% CI = 1.0–2.5). The OR remained significant after controlling for the number of transferred embryos and blastocyst grading (aOR = 1.7; 95% CI = 1.1–2.8). Additionally, blastocyst grading was a significant predictor of LBR (*p* = 0.001); it remained significant after controlling for BMI and number of transferred embryos (*p* < 0.001).

Pregnancy outcome and the corresponding odds ratios of group A and B are summarized in Table 2. Notably, group A exhibited a significantly higher LBR (52.9% vs. 40.1%; *p* = 0.01; OR = 1.6; 95% CI = 1.1–2.5) than group B. The adjusted OR for live birth was 1.7 (95% CI = 1.1–2.8) after controlling for BMI, number of transferred embryos and blastocyst grading (Table 3). Group A exhibited a significantly higher implantation rate (48.7% vs. 38.1%; p = 0.01; OR = 1.5, 95% CI = 1.1–2.1). The odds ratio remained significant after adjusting for blastocyst grading (aOR = 1.6; 95% CI = 1.1–2.3). The spontaneous abortion rate was comparable between the two groups (*p* = 0.8). Group A had a significantly lower progesterone levels on the day of the defined LH surge compared to group B (0.8 [0.7–1.0] vs. 1.0 [0.8–1.2], respectively; *p* < 0.001). However, among women in group A, the LBR was comparable between those with progesterone level ≥ 1 ng/mL and those with progesterone <1 ng/mL (54.1% vs. 52.2%; p = 0.8).

**Table 2:** Univariate analysis of natural cycle frozen-thawed blastocyst transfer outcomes of non-PGS study cohort (*n* = 407)

Parameter	Group A (*n* = 270)	Group B (*n* = 137)	Odds ratio (95% CI)	*P* value
Number of transferred blastocysts	1.5 ± 0.5	1.4 ± 0.5	–	0.50
Clinical pregnancy rate (%)	60.3	46.7	1.7 (1.1–2.6)	0.009
Spontaneous abortion rate (%)	12.2	14.0	0.85 (0.3–1.9)	0.71
Live birth rate (%)	52.9	40.1	1.6 (1.1–2.5)	0.01

Values were expressed as median [interquartile range]

**Table 3:** Odds ratio and adjusted odds ratio for live birth rate after multivariate logistic regression. They were controlled for all variables mentioned in the table

Parameter	Odds ratio (95% CI)	Adjusted odds ratio (95% CI)	Standard error	*P* value
Body mass index^a^				
< 30 kg/m^2^	5.6 (2.3–13.8)	6.6 (2.6–16.8)	3.1	<0.001
Blastocyst grading^b^				
Excellent	–	–	–	<0.001
Good	3.7 (1.3–10.0)	4.5 (1.5–13.2)	2.4	0.006
Average	4.2 (2.0–8.4)	4.9 (2.2–10.8)	1.9	<0.001
Definition of LH surge^c^				
Group A	1.6 (1.1–2.5)	1.7 (1.1–2.8)	0.42	0.01
Number of transferred blastocysts^d^				
2	1.1 (0.7–1.6)	1.1 (0.7–1.8)	0.2	0.4
3	5.7 (0.6–49.7)	3.2 (0.3–33.0)	3.8	0.3

^a^ compared to ≥30 kg/m^2^

^b^ compared to poor embryos

^c^ compared to group B

^d^ compared to 1 blastocyst

### NC-FET of women who underwent PGS (*n* = 284)

A total of 284 NC-FET of blastocysts that underwent PGS (*n* = 247 women) were included. Sixty-two percent were older than 35 years old. Group A and B had comparable IR (57.4% vs. 63%, respectively; *p* = 0.39) and LBR (56.7% vs. 63.4%, respectively; *p* = 0.37) when defining estradiol drop as ≥30%. Similarly, there were no significant differences in IR (57.2% vs. 63%; *p* = 0.34) or LBR (56.3% vs. 63.7%) between group A and B when defining estradiol drop as ≥20%. The two groups had also equivalent IR and LBR at ≥10% drop in estradiol.

## Discussion

The present study was conducted to delineate LH and estradiol levels that best define the LH surge and optimize NC-FET outcome. Our data show that, in non-PGS cycles, considering the first LH of ≥17 IU/L during the follicular phase followed by a ≥30% drop in estradiol level as the LH surge was associated with higher implantation and live birth rates than considering the subsequent higher LH level as the surge. This study also confirms that blastocyst grading and body mass index significantly affect the outcomes of NC-FET in young patients. However, in PGS cycles, there was no difference in the NC-FET outcomes between patients whose LH surge was delineated by either of the two definitions.

During a natural cycle, the endometrium undergoes morphological and biochemical changes that are necessary for implantation. Such changes are mainly mediated by exposure to endogenous estrogen and progesterone. Implantation usually occurs 6–12 days following LH surge [11–14]. The majority of embryos implant 8–10 days post-LH surge, while earlier or later implantations occur more rarely [11]. Interestingly, pregnancies resulting from late implantation (11–12 days following LH surge) are associated with a significant increase in miscarriage rate compared to pregnancies that implant 7–10 days post-LH surge [11, 12, 14, 15]. This could be related to the decrease in endometrial receptivity during the late luteal phase or the decline in the sensitivity of the corpus luteum to human chorionic gonadotropin 11–12 days post-LH surge [15, 16]. Perceived late implantation may also reflect a slow growing embryo, which could be the reason for the observed increase in miscarriages.

In order to achieve an optimal outcome in a NC-FET, transfer of the thawed embryo(s) should be carried out at the time of the highest endometrial receptivity, determined by the time of LH surge. Our findings show that LH and estradiol levels should be re-measured a day after LH ≥ 17 IU/L before determining the LH surge. It is possible that the lower success rate of NC-FET, when defining the LH surge as the highest LH level irrespective of the drop in estradiol levels, is related to the higher rate of embryo-endometrium dyssynchrony due to a relatively longer exposure of the endometrium to progesterone. This theory is supported by the higher progesterone level on the day of the defined LH surge in group B compared to group A. Given that this difference was not observed in PGS cycles, it is possible that embryos which have undergone zona breaching during biopsy implant earlier, thus neutralizing the observed difference in non-hatched embryos [13].

During a natural cycle, the dramatic rise in estradiol levels secreted by the preovulatory follicle induces the LH surge, which in turn stimulates luteinization of granulosa cells and synthesis of progesterone. Thus, the drop in estradiol levels follows the initiation of the LH surge. This explains our findings suggesting that the LH surge is better defined a day before the drop in estradiol level.

The correlation between BMI and IVF outcomes has been well investigated [17–20]. Loveland et al. showed that women with BMI > 25 kg/m^2^ exhibited lower implantation and pregnancy rates and higher spontaneous abortion rate following IVF compared to women with BMI ≤ 25 kg/m^2^ [17]. Wang et al. also reported that the spontaneous abortion rates in women undergoing infertility treatment progressively increased with increasing BMI [18]. Thus, our findings that obesity was associated with lower implantation rates and higher spontaneous abortion rates are similar to those described in the literature [17–21]. It has been suggested that the adverse effects of obesity on IVF outcomes may be attributed to the alteration in the uterine environment [22, 23]. Furthermore, increased levels of inflammatory markers such as interleukin-6 and tumor necrosis factor-α found in obese women may negatively impact implantation and early embryonic development [24–26].

The current study is limited by its retrospective nature. However, to our knowledge, this is the first study to address the definition of LH surge to accurately time embryo transfer during NC-FET.

## Conclusion

In conclusion, measuring LH and estradiol levels a day after the first LH ≥ 17 IU/L during the follicular phase should be performed to better delineate the LH surge and determine the optimal day for embryo transfer in non-PGS NC-FET cycles.

## Abbreviations

AOR: adjusted odds ratio
BMI: body mass index
CI: confidence interval
FET: frozen-thawed embryo transfer cycle
ICM: inner cell mass
IR: implantation rate
LBR: live birth rate
LH: luteinizing hormone
NC-FET: natural cycle frozen-thawed embryo transfer
OHSS: ovarian hyperstimulation syndrome
OR: odds ratio
PGS: preimplantation genetic screening
*χ*^2^: chi-square
